# MicroRNA differential expression analysis in canine visceral hemangiosarcoma formalin-fixed, paraffin-embedded tissues

**DOI:** 10.3389/fvets.2026.1755166

**Published:** 2026-03-17

**Authors:** Laura Machado Ribas, Kerstin Muner, Nelly Elshafie, Elizabeth Rozanski, Francisco O. Conrado, Luis Dos Santos, Andrea Pires dos Santos

**Affiliations:** 1 Department of Veterinary Clinical Sciences, College of Veterinary Medicine, Purdue University, West Lafayette, IN, United States; 2 Purdue Institute for Cancer Research, Purdue University, West Lafayette, IN, United States; 3 Department of Comparative Pathobiology, College of Veterinary Medicine, Purdue University, West Lafayette, IN, United States; 4 Department of Small Animal Clinical Sciences, Cummings School of Veterinary Medicine at Tufts University, North Grafton, MA, United States; 5 Department of Comparative Pathobiology, Cummings School of Veterinary Medicine at Tufts University, North Grafton, MA, United States

**Keywords:** angiosarcoma, cancer biomarkers, miRNAs, pathway analysis, small RNA sequencing, visceral hemangiosarcoma

## Abstract

**Introduction:**

Canine visceral hemangiosarcoma (HSA) is a highly aggressive malignancy of endothelial cells with a poor prognosis and limited diagnostic tools. MicroRNAs (miRNAs) are small non-coding RNAs with emerging utility as diagnostic and prognostic biomarkers due to their stability and disease-specific expression profiles.

**Methods:**

This study aimed to identify miRNA biomarkers for canine splenic and cardiac HSA using small RNA sequencing and quantitative PCR. Formalin-fixed, paraffin-embedded (FFPE) tissues were analyzed from 24 dogs with histologically confirmed HSA (18 splenic, six cardiac) and 12 non-neoplastic controls (six cardiac and six splenic).

**Results:**

A total of 67 and 71 miRNAs were differentially expressed (DE) in splenic and cardiac HSA, respectively, with 18 miRNAs shared between both tumor types. Forty candidate miRNAs were selected for validation by RT-qPCR using customized panels. Thirteen miRNAs were validated as DE in each tissue type, showing strong concordance with sequencing results. Pathway enrichment analysis of validated miRNAs revealed a significant involvement in oncogenic signaling pathways, including the PI3K-Akt, MAPK, HIF-1, and Ras pathways.

**Discussion:**

These results highlight miRNA signatures that may have diagnostic value in visceral HSA and support their use as biomarkers in archived tissues, with potential future application in liquid biopsy approaches.

## 1 Introduction

Canine visceral hemangiosarcoma (HSA) is an aggressive cancer of endothelial cells that is hypothesized to arise from bone marrow-derived endothelial cell precursors (1). It is particularly common in middle-aged to older, large-breed dogs and often affects organs such as the spleen, heart, liver, and lungs (1–4). Its rapid growth, high metastatic rate, and frequent tumor rupture can cause significant internal bleeding, which complicates both clinical presentation and case management (5, 6). Despite advances in treatment, the prognosis for dogs with visceral hemangiosarcoma remains poor. Surgery is the primary treatment, often followed by chemotherapy with doxorubicin; however, the median survival time post-surgery is only around four to six months (7, 8). The prognosis is even worse when metastasis has occurred, with survival rates dropping significantly (9). The gold standard for diagnosis of hemangiosarcoma is through histological analysis of the tumor, which usually reveals irregular blood-filled vascular channels lined by atypical endothelial cells (10). However, diagnosis can be challenging when tumors have atypical presentation, such as in nodular or solid masses without clear vascular formation, requiring thorough microscopic evaluation (11, 12). Additionally, the histologic criteria of malignancy in HSA are highly variable and do not reliably predict prognosis (4). Complementary to histopathology, immunohistochemistry targeting specific endothelial cell markers, such as CD31 and von Willebrand factor, can help differentiate the tumor origin from other conditions like hematoma or other sarcomas (10).

MicroRNAs (miRNAs) play a crucial role in regulating gene expression, influencing processes such as apoptosis, angiogenesis, and tumor growth (13–15). Their stability in formalin-fixed tissues and body fluids, as well as their tissue-specific expression profiles, make miRNAs promising candidates for biomarker development (16–20). Certain miRNAs have been implicated in the disease’s molecular mechanisms in studies on canine hemangiosarcoma. For instance, miR-214 has been identified as a tumor-suppressive miRNA that is downregulated in hemangiosarcoma cells (21). This miRNA promotes apoptosis in neoplastic cells via the COP1-p53 signaling pathway, suggesting its potential utility as a therapeutic agent (13, 22). Another significant miRNA, miR-126, promotes angiogenesis, a crucial aspect of hemangiosarcoma due to its endothelial origin (14, 15). Increased levels of miR-126 have been observed in various cancers, including hemangiosarcoma, indicating a role in tumor progression by enhancing blood vessel formation (14, 15). Additionally, comparative studies have identified alterations in the miRNA expression profile between hemangiosarcoma and normal splenic tissue, underscoring the relevance of miRNAs as potential biomarkers for differentiating malignant from benign lesions (21, 23). Specifically, the expression of miRNAs like miR-452 and miR-494-3p was altered in hemangiosarcoma compared to healthy controls in one study, pointing to their potential as diagnostic markers (23).

In this study, we performed small RNA sequencing and pathway analysis on formalin-fixed paraffin-embedded (FFPE) splenic and cardiac tissues from dogs diagnosed with visceral hemangiosarcoma to identify miRNAs that may be involved in its pathophysiology. Research on canine HSA serves to improve veterinary oncology but also offers valuable insights into the biology of vascular tumors that affect humans, given the similarities in genetic and molecular features between canine and human angiosarcomas (24, 25). This approach may accelerate the development of less invasive diagnostic methods and more effective treatments for these challenging malignancies.

## 2 Materials and methods

### 2.1 Animals

We included archived FFPE tissue samples from 24 dogs with histopathologically confirmed splenic (*n* = 18) and cardiac hemangiosarcoma (*n* = 6) in this study. The inclusion criteria required a survival time greater than five days following hospital discharge. Cases were excluded if the animal was alive at the time of last follow-up or if death was attributed to a cause unrelated to hemangiosarcoma. Control samples consisted of cardiac (*n* = 6) and splenic (*n* = 6) FFPE tissues from dogs that died of non-neoplastic causes and underwent necropsy. All samples (cases and controls) were obtained from the Willie M. Reed Animal Disease Diagnostic Laboratory (ADDL) and were selected from archival submissions within 10 years preceding sample selection (sample metadata in Supplementary Table 1).

### 2.2 RNA isolation

Total RNA was extracted from FFPE tissues using the miRNeasy FFPE Kit (QIAGEN, Germantown, MD), according to the manufacturer’s protocol. Briefly, three 10-μm sections per sample were deparaffinized with xylene, followed by ethanol washes and lysis. RNA was eluted in 26 μL of RNase-free water. RNA quantity was assessed using a NanoDrop^™^ One spectrophotometer (Thermo Fisher Scientific, Waltham, MA) and confirmed with the Qubit RNA High Sensitivity (HS) Assay Kit (ThermoFisher Scientific).

### 2.3 Library preparation and sequencing

#### 2.3.1 Splenic samples

Library preparation and sequencing were performed at QIAGEN using the QIAseq miRNA Library Kit (QIAGEN). A total of 100 ng of input RNA per sample was used to construct small RNA libraries. Unique molecular indices (UMIs) were incorporated during reverse transcription, followed by PCR amplification (16 cycles) and addition of sample indices. Libraries were quality-controlled using capillary electrophoresis (Fragment Analyzer, Agilent Technologies), quantified by quantitative PCR (qPCR), and pooled in equimolar ratios. Sequencing was performed on an Illumina NextSeq 2000 platform using 2 × 75 bp paired-end reads. FASTQ files were generated using Illumina’s bcl2fastq2 software.

#### 2.3.2 Cardiac samples

Small RNA libraries and sequencing were prepared at Theragen Bio (Seongnam-si, Gyeonggi, Republic of Korea) from total RNA samples using the NEXTflex Small RNA-Seq Kit v3 (Bioo Scientific, Austin, TX). Each sample consisted of approximately 100 ng of total RNA input. Adapter ligation was performed using a specific 3′ adapter (sequence: TGGAATTCTCGGGTGCCAAGG), designed to target small RNA molecules. Following adapter ligation, reverse transcription and PCR amplification were performed according to the manufacturer’s instructions to create cDNA libraries enriched for small RNAs. Size selection was conducted to retain fragments corresponding to miRNA molecules (~18–24 nt). The final library pool was quality-controlled using an Agilent Bioanalyzer to confirm fragment size distribution and quantified using Qubit fluorometry. Libraries were sequenced on an Illumina NovaSeq 6000 platform using paired-end 75 bp reads (2 × 75), generating between ~17 and 49 million raw reads per sample.

### 2.4 Read mapping and expression quantification

#### 2.4.1 Splenic samples

Primary analysis was performed using CLC Genomics Server 21.0.4 (QIAGEN). Reads were processed through the “QIAseq miRNA Quantification” workflow, including adapter/UMI trimming and length filtering (<15 nt or >55 nt). Deduplication was based on UMI grouping rules, including sequence similarity correction for singletons. Reads were mapped to miRBase v22.2 for known miRNAs, allowing for isomiR variants (≤2 mismatches or shifted start/end positions). Unaligned reads were secondarily mapped to the *Canis lupus familiaris* reference genome (Ensembl CanFam3.1.101) using the CLC “RNA-Seq Analysis” workflow with default settings.

Differential expression analysis was conducted using the “Empirical analysis of DGE” tool (EdgeR-based) within CLC Genomics Workbench. Variance-stabilizing transformation (VST) was applied using the DESeq2 R package (v1.28.1). *p*-values were adjusted for multiple testing using the Benjamini-Hochberg false discovery rate (FDR) correction. MiRNAs were considered differentially expressed if they exhibited an absolute log_2_ fold change ≥1 and an adjusted *p*-value <0.01.

#### 2.4.2 Cardiac samples

Initial quality control of the raw sequencing reads was performed using FastQC v0.11.9, and adapter trimming was done using Cutadapt v4.1. Reads shorter than 17 nt post-trimming were discarded. On average, over 98% of reads passed the quality and length filters across all samples. High-quality reads were aligned to the CanFam3.1 genome using Bowtie v1.3.1, allowing for efficient and accurate mapping of short reads. Known miRNAs were annotated using miRBase v22.1. Reads were aligned to both mature and precursor miRNA sequences to capture the full spectrum of small RNA species.

Read quantification was performed using HTSeq v2.0.2 to count reads aligned to each annotated miRNA. For differential expression analysis, raw counts were normalized and processed using DESeq2 v1.32.0, employing the VST and negative binomial modeling to identify statistically significant changes in miRNA abundance between biological conditions. Adjusted *p*-values were calculated using the Benjamini-Hochberg procedure to control the FDR. MiRNAs were deemed differentially expressed if they had an absolute log_2_ fold change ≥1 and an FDR-adjusted *p*-value <0.01.

### 2.5 RT-qPCR validation

#### 2.5.1 Normalization strategy

To identify suitable endogenous miRNA normalizers for RT-qPCR validation, we employed a multi-step strategy using our sequencing dataset, as previously described (26). This normalization approach was developed by our research team (26), built upon previously described methods (27, 28). First, miRNAs that were not significantly differentially expressed (FDR >0.05) were filtered. From this subset, miRNAs were ranked by fold change in ascending order and selected those with values closest to one, indicating minimal variation between experimental groups. Next, expression stability was assessed by calculating each candidate’s mean, standard deviation, and coefficient of variation (CV) across all samples using the count-per-million (CPM) matrix. Candidates were ranked by CV, and the top 50% most stable miRNAs were retained. To ensure biological neutrality, any miRNAs previously implicated in cancers were excluded based on a thorough literature review. Three miRNAs (miR-8859a, miR-8859b, and miR-8884) that met all criteria were selected to be tested on the original sequencing cohort. Following RT-qPCR, we evaluated the expression stability of the candidate normalizers using the GeNorm algorithm (29) (Supplementary Tables 2, 3).

#### 2.5.2 Quantitative reverse transcription PCR

To validate the differential expression of miRNAs identified by sequencing in FFPE tissues of splenic and cardiac hemangiosarcoma, 40 candidate miRNAs were selected based on fold-change, statistical significance, and relevance to biological pathways (gene ontology analysis). Customized miRNA qPCR panels were developed (miRCURY LNA miRNA Custom PCR Panel, QIAGEN), incorporating 40 target miRNAs, as well as the three candidate endogenous miRNA controls, RNU6, UniSp3, and UniSp6 (Table 1).

**Table 1:** MicroRNAs selected for customized qPCR plates design.

#	MiRCury Assay Cat. No.	miRNA ID	Target sequence
1	YP00205943	miR-20b	CAAAGUGCUCACAGUGCAGGUA
2	YP02113807	miR-10a	UACCCUGUAGAUCCGAAUUUGU
3	YP02110018	miR-141	AACACUGUCUGGUAAAGAUGG
4	YP02102101	miR-142	CCCAUAAAGUAGAAAGCACUA
5	YP02100678	miR-188	CAUCCCUUGCAUGGUGGAGGGU
6	YP02113947	miR-196b	UAGGUAGUUUCCUGUUGUUGGGA
7	YP02104713	miR-221	AGCUACAUUGUCUGCUGGGUUU
8	YP02116318	miR-32	UAUUGCACAUUACUAAGUUGCAU
9	YP02114619	miR-411	AUAGUAGACCGUAUAGCGUACG
10	YP02115385	miR-503	UAGCAGCGGGAACAGUACUG
11	YP02102538	miR-18a	UAAGGUGCAUCUAGUGCAGAUA
12	YP02100265	miR-18b	UAAGGUGCAUCUAGUGCAGUUA
13	YP00204130	miR-135b	UAUGGCUUUUCAUUCCUAUGUGA
14	YP00204688	miR-146a	UGAGAACUGAAUUCCAUGGGUU
15	YP00204660	miR-150	UCUCCCAACCCUUGUACCAGUG
16	YP00204018	miR-187	UCGUGUCUUGUGUUGCAGCCGG
17	YP00204665	miR-193a	UGGGUCUUUGCGGGCGAGAUGA
18	YP00204482	miR-200c	UAAUACUGCCGGGUAAUGAUGGA
19	YP00205616	miR-202	UUCCUAUGCAUAUACUUCUUUG
20	YP00205914	miR-203a	GUGAAAUGUUUAGGACCACUAG
21	YP00204487	miR-205	UCCUUCAUUCCACCGGAGUCUG
22	YP00205401	miR-212	ACCUUGGCUCUAGACUGCUUACU
23	YP00204364	miR-328	CUGGCCCUCUCUGCCCUUCCGU
24	YP02119293	miR-335	UCAAGAGCAAUAACGAAAAAUGU
25	YP00204486	miR-34a	UGGCAGUGUCUUAGCUGGUUGU
26	YP00205659	miR-34c	AGGCAGUGUAGUUAGCUGAUUGC
27	YP00204618	miR-362	AAUCCUUGGAACCUAGGUGUGAGU
28	YP00204011	miR-370	GCCUGCUGGGGUGGAACCUGGU
29	YP00204362	miR-375	UUUGUUCGUUCGGCUCGCGUGA
30	YP00204218	miR-376b	AUCAUAGAGGAAAAUCCAUGUU
31	YP00204301	miR-452	AACUGUUUGCAGAGGAAACUGA
32	YP00204489	miR-487b	AAUCGUACAGGGUCAUCCACUU
33	YP00204579	miR-494	UGAAACAUACACGGGAAACCUC
34	YP00205657	miR-505	GGGAGCCAGGAAGUAUUGAUGU
35	YP00204447	miR-543	AAACAUUCGCGGUGCACUUCUU
36	YP00205983	miR-99b	CACCCGUAGAACCGACCUUGCG
37	YP00205427	miR-376c	GUGGAUAUUCCUUCUAUGUUUA
38	YP02119694	miR-7a	UGGAAGACUAGUGAUUUUGUUGU
39	YP00205120	miR-223	UGUCAGUUUGUCAAAUACCCC
40	YP00205141	miR-493	UGAAGGUCUACUGUGUGCCAG
41	YP00203907	U6 snRNA	
42	YP02119288	UniSP3	
43	YP00203954	UniSp6	
44	YP02121094	miR-8859a	UGGAUCGGAGCCGGGGUCCGGA
45	YP02121097	miR-8859b	GGUCGGAUUCCGUGCCUGGAGU
46	YP02121124	miR-8884	UUUGAUGGAUUUGCUUAGCACC

First-strand cDNA was synthesized by reverse transcription using 50 ng of total RNA and the miRCURY LNA RT Kit (QIAGEN), according to the manufacturer’s instructions. The synthetic RNA spike-in UniSp6 was included as an internal control. qPCR was performed using the miRCURY LNA SYBR Green PCR Kit (QIAGEN) following the recommended protocol. For analysis, CT values were uploaded to the QIAGEN GeneGlobe Data Analysis Center.^1^ Samples were assigned to control and test groups, and normalization was performed using the GeNorm method (29) based on our predefined reference miRNAs. Relative expression levels were calculated using the ∆∆CT method, where the ∆CT of each target was computed relative to the average of selected reference miRNAs, and the ∆∆CT was calculated by subtracting the ∆CT of the control group from that of the test group. Fold change was calculated using the 2^(−∆∆CT)^ formula.

### 2.6 Pathway enrichment analysis

MiRNAs significantly differentially expressed in the small RNA sequencing analysis and successfully validated by quantitative reverse transcription PCR (RT-qPCR) were selected for pathway analysis. Predicted target genes for each validated miRNA were identified using miRDB,^2^ a web-based tool that provides functional annotations for miRNA targets based on a machine learning algorithm trained on high-confidence experimental data (30, 31). For each miRNA, the top-ranked predicted targets with a target score ≥60 were extracted from miRDB. The compiled list of target genes was then uploaded to ShinyGO^3^ (32), an interactive gene-set enrichment analysis tool, to identify enriched Gene Ontology (GO) terms and Kyoto Encyclopedia of Genes and Genomes (KEGG) pathways (33, 34). Enrichment analysis was performed with default parameters, using the *Canis lupus familiaris* genome (gene symbol-based annotation on ROS_Cfam_1.0 GCF_014441545.1) as the background.

## 3 Results

### 3.1 Profiling of differentially expressed microRNAs in tissues with hemangiosarcoma

**Figure 1:**
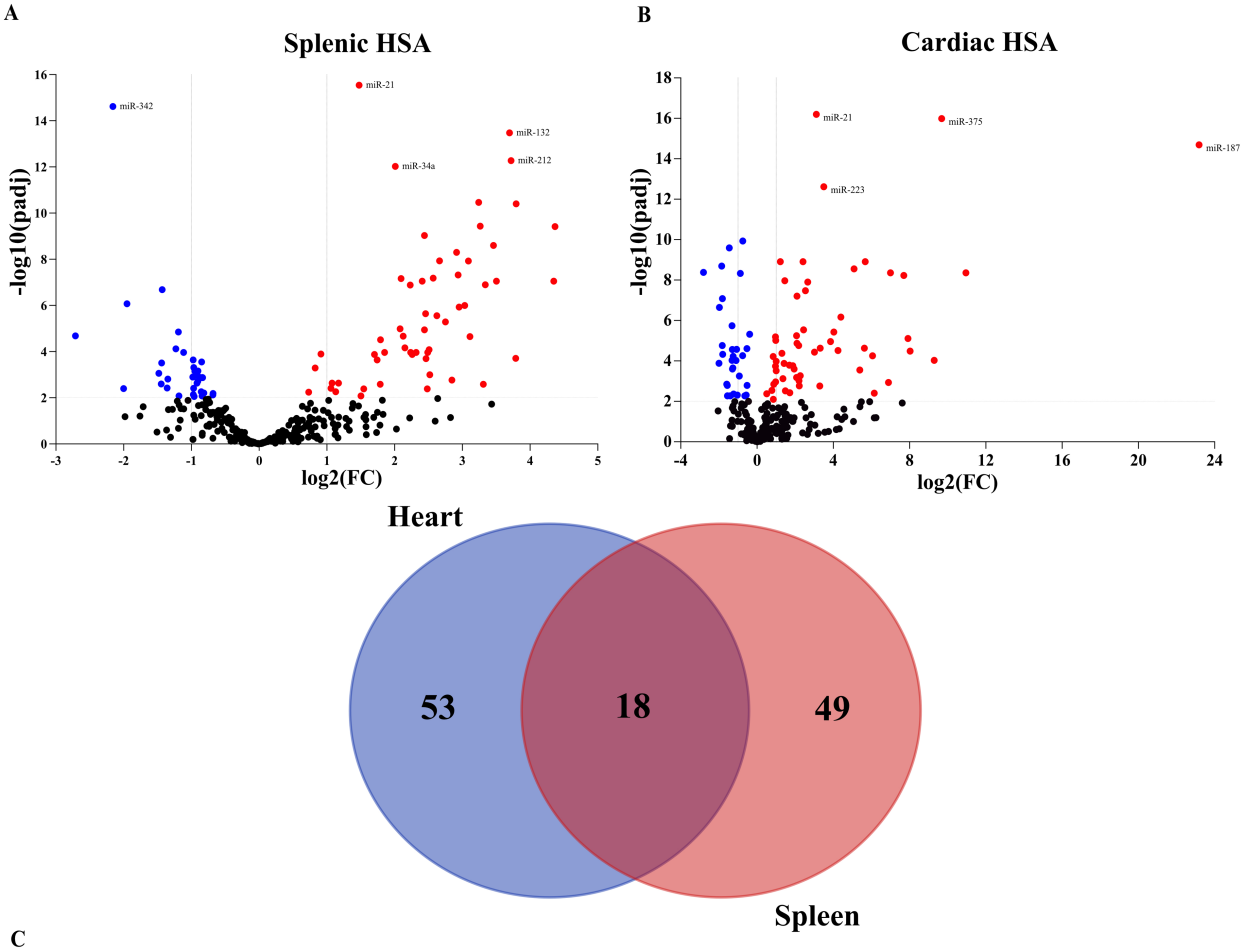
Differential expression analysis of microRNAs in visceral hemangiosarcoma via small RNA sequencing. **(A,B)** Volcano plot identifying miRNA expression changes. The *x*-axis represents the log_2_ of the fold changes in miRNA expression, whereas the *y*-axis shows the statistical significance. The two center vertical lines indicate a 2-fold regulation threshold. The horizontal line indicates a 0.01 *p*-value threshold. Red represents positive regulation. Blue represents negative regulation. **(C)** Venn diagram indicating the number of overlapping and unique miRNAs between the two datasets.

To determine if miRNAs are altered in tissues with HSA, we performed small RNA sequencing on 18 splenic hemangiosarcoma samples and six splenic controls. A total of 453 miRNAs were detected across all samples. Differential expression analysis identified 67 miRNAs significantly dysregulated in splenic HSA compared to normal spleen, using a threshold of ≥2-fold change and FDR <0.01 (Figure 1A and Supplementary Table 4). The distribution of expression patterns suggested distinct molecular signatures related to malignant endothelial transformation in splenic tissue.

To assess organ-specificity, small RNA sequencing was also conducted on cardiac hemangiosarcoma tissues. This analysis revealed 71 differentially expressed miRNAs in cardiac HSA using the same criteria of ≥2-fold change and FDR <0.01 (Figure 1B and Supplementary Table 5). Of these, 53 miRNAs were uniquely dysregulated in cardiac tissue, not overlapping with those identified in splenic samples. Conversely, 18 miRNAs were shared between cardiac and splenic HSA tissues, indicating tissue-specific and common regulatory patterns across visceral HSA sites (Figure 1C and Supplementary Table 6). The miRNAs dysregulated in cardiac samples were enriched for functions in vascular development, endothelial migration, immune regulation, and angiogenic signaling, reflecting the aggressive biology of cardiac HSA and its endothelial origin.

### 3.2 Validation of differentially expressed miRNAs

**Figure 2:**
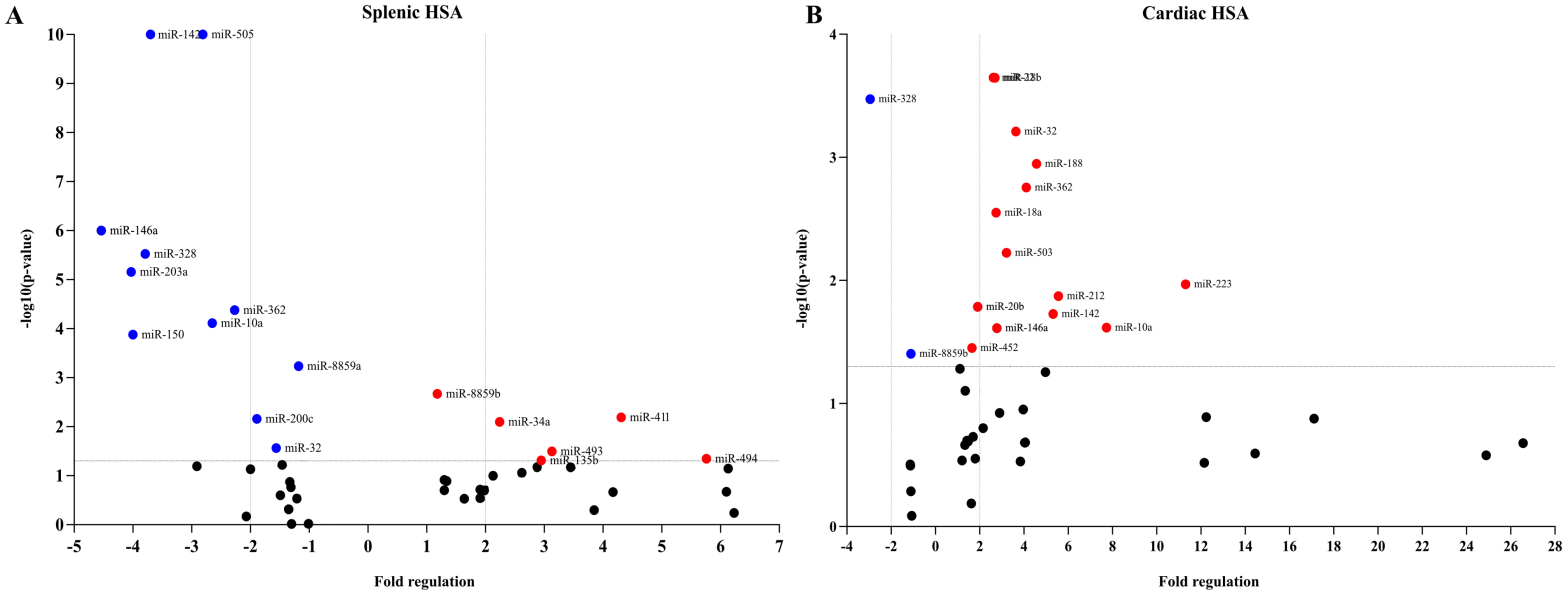
Volcano plot of RT-qPCR validation analysis of microRNA candidates for diagnostic markers for visceral hemangiosarcoma. The *x*-axis represents the fold regulations in miRNA expression, whereas the *y*-axis shows the statistical significance. The two center vertical lines indicate a 2-fold regulation threshold. The horizontal line indicates a 0.05 *p*-value threshold. Red represents positive regulation. Blue represents negative regulation. **(A)** Splenic hemangiosarcoma. **(B)** Cardiac hemangiosarcoma.

A customized qPCR panel was developed to validate miRNA candidates identified by small RNA sequencing, including 40 differentially expressed miRNAs and six controls. These miRNAs were selected based on sequencing fold change, statistical significance, and known or predicted involvement in tumor-related pathways. Validation was performed using RNA extracted from FFPE splenic hemangiosarcoma tissues. Of the 40 tested miRNAs, 13 were confirmed as significantly differentially expressed in splenic hemangiosarcoma compared to healthy spleen tissue. Among these, five miRNAs were upregulated, and eight were downregulated, showing consistent directionality with the sequencing data (Figure 2A and Table 2). These validated miRNAs further support their potential as biomarkers for distinguishing splenic HSA from normal tissue.

**Table 2:** Differentially expressed miRNAs in splenic hemangiosarcoma.

miRNA ID	Fold regulation	*p*-value
miR-34a	2.24	0.007977
miR-135b	2.95	0.048684
miR-493	3.13	0.031991
miR-411	4.31	0.006456
miR-494	5.76	0.045127
miR-10a	−2.65	0.000077
miR-142	−3.70	0.000000
miR-146a	−4.54	0.000001
miR-328	−3.79	0.000003
miR-505	−2.81	0.000000
miR-150	−4.00	0.000133
miR-362	−2.27	0.000042
miR-203a	−4.03	0.000007

The same panel of 40 miRNAs was analyzed by qPCR in FFPE cardiac hemangiosarcoma samples. Thirteen miRNAs were significantly dysregulated in cardiac HSA compared to control cardiac tissue. Notably, 12 miRNAs were upregulated, and one was downregulated in tumor tissue, aligning with the patterns observed in the sequencing dataset (Figure 2B and Table 3). The validated miRNAs included both unique cardiac-specific candidates and miRNAs shared with the splenic hemangiosarcoma subset, showing both organ-specific and overlapping molecular features of visceral HSA. The concordance between qPCR and sequencing results supports the robustness of the selected miRNA markers and their potential clinical utility.

**Table 3:** Differentially expressed miRNAs in cardiac hemangiosarcoma.

miRNA ID	Fold regulation	*p*-value
miR-10a	7.73	0.024233
miR-18a	2.74	0.002813
miR-142	5.32	0.018751
miR-503	3.21	0.005969
miR-146a	2.78	0.024394
miR-221	2.62	0.000225
miR-212	5.56	0.013431
miR-18b	2.69	0.000226
miR-32	3.64	0.000616
miR-188	4.57	0.001131
miR-362	4.11	0.001758
miR-223	11.31	0.010768
miR-328	−2.95	0.000337

### 3.3 Pathway enrichment analysis

**Figure 3:**
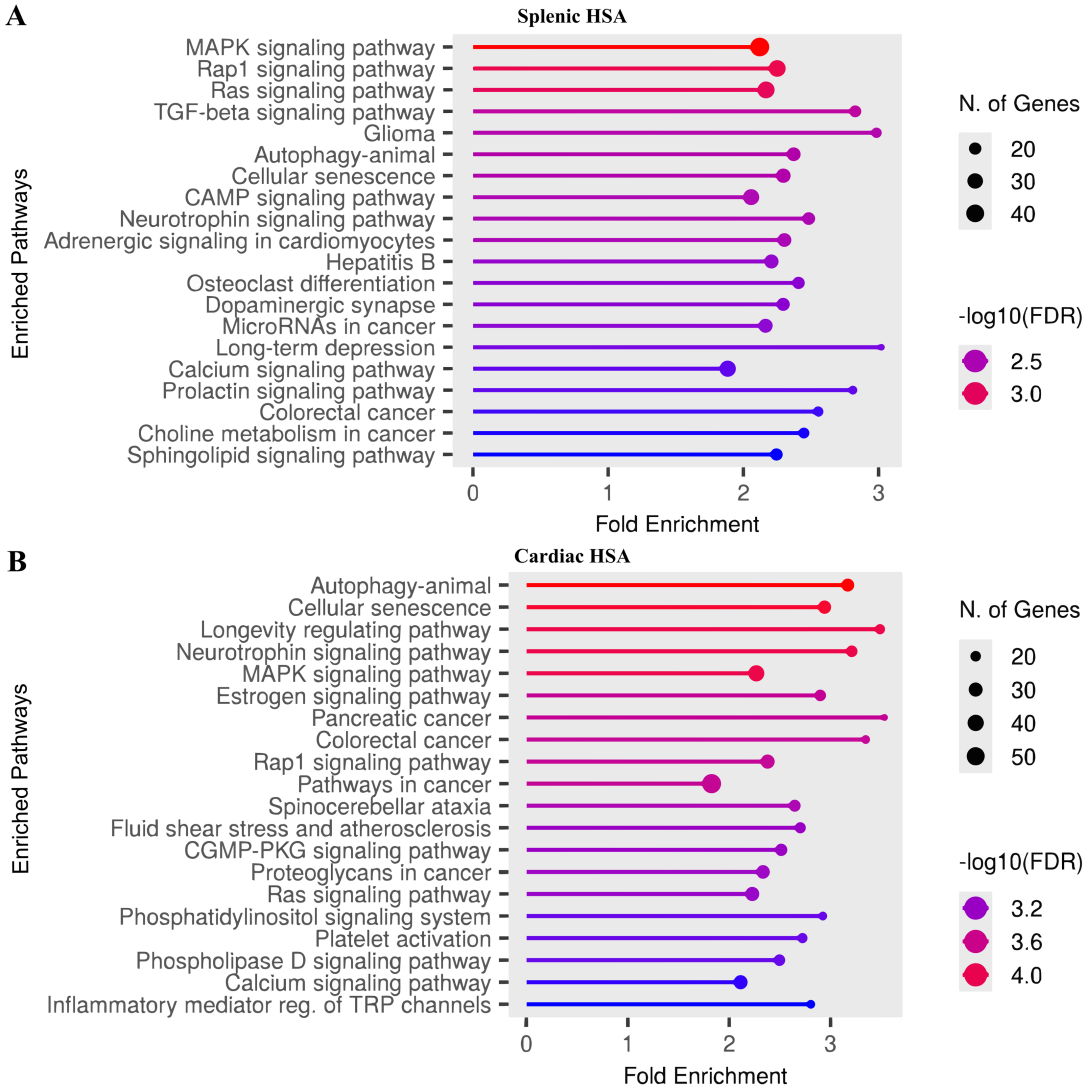
KEGG pathway enrichment analysis based on target genes of differentially expressed miRNAs in visceral hemangiosarcoma. The *y*-axis lists the enriched pathways, while the *x*-axis represents fold enrichment values. **(A)** Splenic hemangiosarcoma. **(B)** Cardiac hemangiosarcoma.

To explore the biological roles of differentially expressed miRNAs in splenic HSA, we performed pathway enrichment analysis using ShinyGO with the target genes predicted by Target Mining on miRDB (Supplementary Tables 7, 8). The study revealed significant enrichment in various cancer-related and signaling pathways (Figure 3A). The top enriched pathways included the MAPK, Rap1, and Ras signaling pathways, which are well-known mediators of tumor cell proliferation, differentiation, and survival (Supplementary Figures 1–3, respectively). Next was the TGF-β signaling pathway, an important regulator of tumor progression, angiogenesis, and immune evasion within the tumor microenvironment (Supplementary Figure 4). Other enriched pathways included autophagy, cellular senescence, and glioma, suggesting overlapping mechanisms with different aggressive and vascular-rich tumor types. Notably, pathways involved in neuroactive signaling, such as neurotrophin signaling and dopaminergic synapse, were also identified, which may reflect interactions between blood vessel formation and neural pathways in the tumor niche. Cancer-specific pathways, including miRNAs in cancer, colorectal cancer, and choline metabolism in cancer, were also enriched, further reinforcing the oncogenic relevance of the miRNA signature.

Upon pathway enrichment analysis of differentially expressed miRNAs in cardiac HSA, we identified a broad range of enriched pathways (Figure 3B). Top enriched pathways included autophagy, cellular senescence, and the longevity regulating pathway, suggesting roles in tumor cell survival, stress response, and resistance to apoptosis (Supplementary Figures 5–7, respectively). Key oncogenic signaling pathways such as MAPK, Rap1, Ras, and estrogen signaling were also significantly enriched, highlighting critical mechanisms that drive proliferation, angiogenesis, and cellular transformation in endothelial-derived tumors (Supplementary Figure 8). In addition, several cancer-related KEGG pathways were overrepresented, including those associated with pancreatic, colorectal, and general pathways in cancer, suggesting an association between the miRNA signature and malignancy. Other notable enriched pathways included fluid shear stress and atherosclerosis, platelet activation, and phosphatidylinositol signaling, all of which are closely linked to vascular function and hemodynamic regulation, key aspects of cardiac HSA biology. The enrichment of neurotrophin signaling and inflammatory mediator regulation of TRP channels also suggests a possible interaction between neural, inflammatory, and vascular cues in the tumor microenvironment. Overall, these results highlight that the dysregulated miRNAs in HSA are functionally linked to key signaling cascades involved in tumorigenesis, vascular remodeling, and immune modulation.

## 4 Discussion

This study provides a comprehensive evaluation of miRNA expression profiles in canine visceral HSA, with particular attention to the differences between splenic and cardiac tumors. We used small RNA sequencing and verified our findings with qPCR, confirming 13 differentially expressed miRNAs in each organ. Among these, miR-328 was the only miRNA consistently downregulated in both tissue types. Four miRNAs (miRs- 142, 10a, 362, and 146a) were dysregulated in both tissue types, though in different directions of expression. In addition, in cardiac HSA, miRs- 32, 18a, 503, 188, 223, 221, 18b, and 212 were significantly upregulated. In contrast, splenic HSA exhibited upregulation of miRs- 493, 494, 34a, 135b, and 411, along with downregulation of miRs- 150, 505, and 203a. While these patterns suggest organ-associated differences in miRNA dysregulation, they should be interpreted cautiously given the use of different sequencing platforms and analytical pipelines across tissues, making our standardized validation step crucial for confirmation. These findings contribute to a growing body of evidence that miRNAs play critical roles in the pathogenesis, progression, and potential prognosis of HSA, with implications for both veterinary and human oncology.

Recent computational and multi-omics studies emphasize that robust biomarkers often reflect coordinated molecular networks rather than single features (35–38). This framework is relevant to the tissue-associated differences observed here, as cardiac and splenic HSA likely arise within distinct molecular and stromal environments that may contribute to divergent miRNA dysregulation patterns. Integrative network modeling could therefore be leveraged in future comparative studies to test whether the miRNA signatures identified in canine visceral HSA align with conserved regulatory networks across HSA contexts (38).

Pathway enrichment analysis linked differentially expressed miRNAs in splenic HSA to Ras/MAPK and PI3K-Akt signaling, consistent with prior reports in other systems. For example, miR-34a, which was upregulated in splenic HSA, has been shown in non-canine cancer cell lines and cardiovascular mouse models to modulate components of the Ras-ERK and PI3K-Akt cascades, with reported effects on cell proliferation, angiogenic effects, and endothelial cell function (39, 40). Notably, these effects are highly context dependent: in some models, ectopic miR-34a reduced basal ERK and Akt phosphorylation, consistent with suppression of mitogenic activity (40). In endothelial cells, miR-34a overexpression suppressed HIF-1α and VEGF, impairing angiogenic tube formation and promoting cellular senescence (39). We speculate that miR-34a upregulation may reflect a stress- or feedback-associated modulation of angiogenic and proliferative pathways. Another miRNA upregulated in splenic HSA, miR-135b, has been reported to influence HIF-1α-associated signaling. In a squamous cell carcinoma mouse model, it targeted a HIF-1α upstream inhibitor with associated increases in VEGF expression and microvessel density, consistent with altered HIF-1α-associated angiogenic signaling (41). In the present study, the upregulation of miR-135b in splenic HSA, together with enrichment of HIF-1 and MAPK signaling pathways, suggests association with hypoxia-responsive angiogenic networks.

Multiple HSA-associated miRNAs have been linked to the regulation of the PI3K/Akt pathway by targeting its negative regulators. In cardiac HSA, miR-221 is strongly upregulated; this oncomiR (often in concert with miR-222) has been reported to enhance PI3K/Akt signaling by silencing PTEN, thereby promoting tumor resistance to adriamycin, as demonstrated in human breast cancer tissue and the MCF-7/ADR cell line (42). Upregulation of miR-221/222 has also been reported to target the CDK inhibitors p27^Kip1^ in HeLa cells and p57^Kip2^ in Hep3b, SNU638, and SNU449 cells, thereby accelerating G1/S progression (43, 44). Although the present study did not directly assess PTEN expression, Akt/mTOR activation, or cell-cycle regulation, enrichment of PI3K/Akt-related pathways in cardiac HSA offers insight into the observed miR-221 dysregulation. Accordingly, the upregulation of miR-221 in cardiac HSA should be interpreted as an association consistent with prior reports linking this miRNA to survival and proliferative signaling, and as a candidate regulatory axis warranting targeted functional validation in canine HSA (42, 45). Similarly, miR-32 and miR-494, both upregulated in cardiac and splenic HSA, respectively, are also reported to target PTEN. Overexpression of miR-32 has been shown to reduce PTEN in SW480 cells and PHLPP2 in ZR-75-30 cells, two inhibitors of the PI3K/Akt pathway, increasing Akt phosphorylation and downstream mTOR activity (46, 47). Whereas miR-494 repressed PTEN in lung cancer A549 cells and liver cancer AML12 cells, leading to hyperactivation of Akt and downstream mTOR/S6K signaling (48, 49). Moreover, miR-494 is part of the 14q32 miRNA cluster often associated with cancer stemness and angiogenesis; its high expression in tumors has been linked to invasive, stem-like phenotypes (48). Target genes of miR-494 also include Sox7 (50), BIM (51), and caspase-2 (52), further illustrating its pro-tumor activity. Collectively, these miRNAs represent candidate regulators of PI3K/Akt-associated signaling networks in canine HSA and provide a rationale for future functional studies aimed at defining their specific roles in tumor biology.

In contrast to miRNAs previously associated with enhanced proliferative and survival, other dysregulated miRNAs identified in cardiac HSA have been linked in prior studies to growth-inhibitory or anti-angiogenic functions. Among these, miR-503, which was upregulated in cardiac HSA, is part of the miR-424/503 cluster, a hypoxia-responsive miRNA locus that has been reported to exert context-dependent, and often tumor-suppressive, effects in several experimental systems (53, 54). In non-canine cell models, miR-503 has been shown to influence cell-cycle regulation by targeting Cyclin D1/3 and E2F3, resulting in G1 arrest in endometriotic stromal cells and hepatocellular carcinoma cell lines (55, 56). Notably, Zhou et al. (57) found that, in HepG2 cells, miR-503 simultaneously downregulates VEGFA and FGF2, two major angiogenic signals, leading to impaired capillary tube formation by human umbilical vein endothelial cells (HUVECs). The upregulation of miR-503 is best interpreted as a compensatory response to excessive angiogenic drive by lowering VEGF/FGF and slowing cell-cycle progression rather than a defined functional role.

Cellular senescence and autophagy were identified among the biological processes associated with the differentially expressed miRNAs in this study. One such miRNA, miR-146a, which was downregulated in splenic HSA and upregulated in cardiac HSA, has been reported in other experimental systems to modulate inflammatory signaling. In a human embryonic kidney (293) cell line, miR-146a was shown to target the adaptor kinases IRAK1 and TRAF6, resulting in attenuation of NF-κB signaling and reduced downstream cytokine secretion (58). In addition, prior studies have shown that repression of IRAK1 can influence the senescence-associated secretory phenotype (SASP) (59). In the present study, differential expression of miR-146a between splenic and cardiac tumors suggests tissue-associated differences in inflammatory or stress-related regulatory networks. A similar context-dependent pattern was observed for miR-10a, which was downregulated in splenic HSA and upregulated in cardiac HSA. This miRNA has been linked in other systems to endothelial activation, inflammation, and senescence. In endothelial cell models, knockdown of miR-10a has been shown to enhance NF-κB signaling through upregulation of key mediators of IκB degradation, including MAP3K7 (TAK1) and βTRCP, with associated increases in VCAM-1, IL-6, and IL-8 expression (60). Conversely, enforced miR-10a expression reduced basal VCAM-1 and E-selectin levels, consistent with attenuation of endothelial inflammatory activation (60). Thus, it is possible that cardiac HSA’s upregulation of miR-10a could temper such pathways in that context, possibly related to different hemodynamic or hypoxic conditions in cardiac tumors.

Consistent with the enrichment of autophagy and stress-related pathways in cardiac HSA, miR-212 was upregulated in cardiac HSA. In a mouse cardiomyocyte model, Ucar et al. (61) reported that miR-212/132 downregulates FoxO3, modulating autophagy and promoting hypertrophy. In cancer, FoxO3a acts as a tumor suppressor by triggering apoptosis or cell-cycle arrest under stress (62). We speculate that miR-212 upregulation in cardiac HSA may suppress FoxO3-driven autophagic cell death, allowing tumor cells to survive under conditions of nutrient deprivation or hypoxia. It aligns with the enrichment of autophagy and mTOR pathways observed, as miR-212 would tilt the balance toward mTOR (growth) and away from FoxO3 (catabolic autophagy). Another reported regulator of the FoxO pathway is miR-362, which was downregulated in splenic HSA but upregulated in cardiac HSA. Zhu et al. (63) reported in 786-O and ACHN renal cell carcinoma cell lines that miR-362-3p was associated with reduced proliferation and epithelial-to-mesenchymal transition through direct targeting of SP1, with downstream effects on PTEN and FoxO3 signaling. In cervical cancer cell lines, SP1 overexpression has been linked to increased cellular proliferation (64), providing biological context for the potential relevance of this axis, where miR-362 loss may promote a more proliferative, invasive phenotype in splenic HSA. Conversely, the upregulation of miR-362 in cardiac HSA could indicate enforcement of a more quiescent state via SP1/PTEN/Akt modulation. While biologically plausible, the functional relevance of this axis in HSA remains to be determined. Finally, miR-203a (downregulated in splenic HSA) has been implicated in regulation of cellular senescence and stemness-related pathways in other tumor models, reinforcing the theme of senescence loss. Notably, miR-203 has been shown to target the polycomb group protein BMI1 in colon cancer cells and leukemia stem cells (65, 66), a master regulator of stem cell self-renewal that extends cellular lifespan through repression of the INK4A/ARF locus (66). In addition, restoration of miR-203 in human T-cell tumor cell lines reduced ABL1 expression and was associated with decreased cellular proliferation (67). In the context of the present study, reduced miR-203a expression in splenic HSA is consistent with dysregulation of BMI1-INK4A/ARF-associated senescence pathways. Notably, similar patterns of miR-203 downregulation have been reported in human soft tissue sarcomas, where they are associated with more aggressive disease and poorer outcomes (68), further supporting the biological relevance of this miRNA in mesenchymal tumors.

Given the central role of aberrant vascular growth in HSA, we next highlight additional dysregulated miRNAs with reported links to angiogenic signaling. MiR-411, which was upregulated in splenic HSA, has been reported to modulate angiogenic and growth factor-responsive pathways in a context-dependent manner. In non-small cell lung cancer models, miR-411 has been shown to target SPRY4, a negative regulator of Ras/ERK signaling, resulting in enhanced EGFR-, ERK-, and AKT-associated signaling and increased invasive behavior (69, 70). Interestingly, while miR-411 appears oncogenic in lung cancer, in other contexts, such as glioblastoma and RCC tissues and cell lines (71, 72), it has been reported as a tumor suppressor, illustrating cell-type specificity. In the context of a highly vascular tumor such as HSA, upregulation of miR-411 is consistent with a role in sustaining angiogenic signaling. Conversely, miR-328, which was consistently downregulated in both splenic and cardiac HSA, has been described in multiple systems as an angiogenesis-inhibitory miRNA. MiR-328 has been shown to target CD44, a cell-surface receptor involved in extracellular matrix interactions, growth factor presentation, and endothelial cell adhesion and migration (73–75). In endothelial and hypoxia-associated human and murine models, reduced miR-328 expression has been associated with enhanced angiogenic signaling (76), including activation of AKT/mTOR and HIF-1α-VEGF-related pathways (77). Accordingly, loss of miR-328 in HSA is consistent with removal of inhibitory constraints on neovascularization. A related pattern is observed for miR-505, which was downregulated in splenic HSA and has been reported in other cancers to target growth factor ligands and kinases within EGFR-Ras-MAPK signaling pathways, including TGFA and MAP3K3 (78, 79). Collectively, these miRNA expression patterns support the presence of a pro-angiogenic regulatory landscape in HSA, characterized by the downregulations of inhibitory “angiomiRs” and altered modulation of growth factor-responsive pathways.

This study has limitations that should be considered when interpreting the results. The cardiac HSA cohort was relatively small, limiting statistical power and rendering those findings exploratory. In addition, differences in sequencing platforms and analytical pipelines between splenic and cardiac samples limit interpretation of tissue-associated differences despite harmonized RT-qPCR validation. Analyses were performed on whole tumor tissue, capturing both tumor-intrinsic and microenvironmental signals, and therefore do not allow attribution of miRNA changes to specific cellular compartments. Prior chemotherapy was not used as an exclusion criterion, and animals were not stratified by treatment status; because chemotherapy can modulate miRNA expression through effects on apoptosis, cellular stress, and immune pathways, treatment-related molecular alterations may have contributed to the observed expression profiles. Dogs with hemangiosarcoma were also significantly older than controls, and age-associated changes in miRNA expression were not included as covariates in the statistical models, potentially introducing a confounding effect. The inclusion criterion requiring a survival time greater than five days after hospital discharge may introduce a survival bias, as it excludes per acute fatal cases. This threshold was established to ensure that only animals with confirmed post-surgical survival and complete follow-up data were included, thereby reducing confounding effects from perimortem physiological stress or acute postoperative complications. Finally, pathway enrichment and target prediction analyses were based on *in silico* approaches, as direct assessment of target gene expression or pathway activation was beyond the scope of this study. Collectively, these factors warrant cautious interpretation and support the need for larger, cell-type-resolved, and functionally focused follow-up studies.

In conclusion, this study defines complex yet coherent regulatory network patterns of miRNA dysregulation in canine visceral HSA that are associated with biological processes central to vascular tumor biology, including angiogenesis, inflammatory signaling, cell-cycle regulation, and cellular stress responses. Pathway enrichment analyses highlight coordinated involvement of PI3K/Akt and MAPK signaling networks, with multiple differentially expressed miRNAs converging on shared regulatory nodes such as PTEN, KRAS, SPRY4, and FOXO3. Tissue-associated differences in miRNA expression provide a framework linking miRNA profiles to pathway-level alterations in HSA. Future studies should prioritize functional validation and evaluation against clinically relevant differential diagnoses (including hematoma, nodular hyperplasia, hemangioma, and other sarcomas) to establish biological and diagnostic relevance, with integration of these data informing translational applications in vascular tumors. Because tumor cells actively and passively release miRNAs into the bloodstream through exosomes, microvesicles, apoptotic bodies, or protein-bound complexes, prospective studies evaluating these candidate miRNAs in matched plasma or serum samples from dogs with cardiac and splenic masses, including benign differentials such as chemodectoma, hematoma or nodular hyperplasia, should be performed to determine their true translational utility.

## Data Availability

The datasets generated for this study have been deposited in NCBI’s Gene Expression Omnibus (80, 81) and are accessible through GEO Series accession number GSE310480 (https://www.ncbi.nlm.nih.gov/geo/query/acc.cgi?acc=GSE310480).

## Footnotes

1 www.qiagen.com/geneglobe

2
http://www.mirdb.org/

3
http://bioinformatics.sdstate.edu/go/

## Supplementary material

SUPPLEMENTARY FIGURE 1 KEGG MAPK signaling pathway enrichment of differentially expressed splenic hemangiosarcoma miRNAs. KEGG pathway map of the MAPK signaling cascade displaying predicted gene targets of significantly differentially expressed miRNAs identified in splenic hemangiosarcoma by DESeq2 analysis (adjusted *p*-value <0.05). Target genes predicted using miRDB are highlighted in red.

SUPPLEMENTARY FIGURE 2 KEGG Rap1 signaling pathway enrichment of splenic hemangiosarcoma-associated miRNAs. KEGG pathway diagram of the Rap1 signaling pathway showing predicted mRNA targets (red) of significantly dysregulated splenic hemangiosarcoma miRNAs. Predicted targets were generated using miRDB.

SUPPLEMENTARY FIGURE 3 KEGG Ras signaling pathway enrichment of splenic hemangiosarcoma-associated miRNAs. KEGG Ras signaling pathway map illustrating predicted mRNA targets (red) of differentially expressed splenic hemangiosarcoma miRNAs.

SUPPLEMENTARY FIGURE 4 KEGG TGF-β signaling pathway enrichment of splenic hemangiosarcoma-associated miRNAs. KEGG pathway representation of the TGF-β signaling cascade showing predicted targets (red) of significantly dysregulated splenic miRNAs. Both SMAD-dependent and SMAD-independent branches are depicted.

SUPPLEMENTARY FIGURE 5 KEGG autophagy pathway enrichment of cardiac hemangiosarcoma-associated miRNAs. KEGG autophagy pathway map displaying predicted mRNA targets (red) of significantly differentially expressed cardiac hemangiosarcoma miRNAs identified by DESeq2. Predicted interactions were obtained from miRDB.

SUPPLEMENTARY FIGURE 6 KEGG cellular senescence pathway enrichment of cardiac hemangiosarcoma-associated miRNAs. KEGG cellular senescence pathway showing predicted targets (red) of dysregulated cardiac hemangiosarcoma miRNAs.

SUPPLEMENTARY FIGURE 7 KEGG longevity regulating pathway enrichment of cardiac hemangiosarcoma-associated miRNAs. KEGG longevity regulating pathway map illustrating predicted targets (red) of significantly differentially expressed cardiac miRNAs.

SUPPLEMENTARY FIGURE 8 KEGG estrogen signaling pathway enrichment of cardiac hemangiosarcoma-associated miRNAs. KEGG estrogen signaling pathway diagram showing predicted gene targets (red) of differentially expressed cardiac hemangiosarcoma miRNAs. Both genomic and non-genomic estrogen receptor signaling components are represented, including interactions with MAPK and PI3K pathways.

SUPPLEMENTARY TABLE 1 Clinical and demographic characteristics of enrolled dogs. Summary of case and control subjects included in the study. Variables include diagnosis, breed, date of birth, age at diagnosis, sex, body weight at diagnosis, presence of gross metastasis, and chemotherapy status. These data describe the clinical population used for RNA sequencing and qPCR validation analyses.

SUPPLEMENTARY TABLE 2 Splenic sample qPCR normalization and reference miRNA stability analysis. Raw cycle threshold (Ct) values and stability assessment for candidate reference miRNAs used in splenic samples. Stability factors were calculated to determine optimal reference miRNAs for normalization. Arithmetic mean and averaged normalization values used for relative expression analysis are provided.

SUPPLEMENTARY TABLE 3 Cardiac sample qPCR normalization and reference miRNA stability analysis. Raw Ct values and stability assessment for candidate reference miRNAs in cardiac samples. Stability factors were used to select reference miRNAs for normalization. Arithmetic mean and averaged normalization values applied in downstream relative quantification are reported.

SUPPLEMENTARY TABLE 4 Differential expression analysis of splenic hemangiosarcoma miRNAs (DESeq2). Results of RNA-sequencing differential expression analysis comparing splenic hemangiosarcoma samples to non-neoplastic splenic controls using DESeq2. The table includes miRNA identifier, log_2_ fold change (log_2_FC), and Benjamini–Hochberg adjusted *p*-value (*p*_adj_). miRNAs meeting statistical significance criteria (*p*_adj_ < 0.05) were considered differentially expressed.

SUPPLEMENTARY TABLE 5 Differential expression analysis of cardiac hemangiosarcoma miRNAs (DESeq2). Results of RNA-sequencing differential expression analysis comparing cardiac hemangiosarcoma samples to non-neoplastic cardiac controls using DESeq2. The table includes miRNA identifier, log_2_ fold change (log_2_FC), and Benjamini–Hochberg adjusted *p*-value (*p*_adj_). miRNAs with *p*_adj_ < 0.05 were considered significantly differentially expressed.

SUPPLEMENTARY TABLE 6 Comparison of differentially expressed miRNAs between splenic and cardiac hemangiosarcoma. Venn comparison of significantly differentially expressed miRNAs identified in splenic and cardiac hemangiosarcoma tissues. The table lists miRNAs unique to splenic tumors, unique to cardiac tumors, and shared between both anatomical locations.

SUPPLEMENTARY TABLE 7 miRDB-predicted mRNA targets of differentially expressed splenic hemangiosarcoma miRNAs. Predicted mRNA targets generated using the miRDB database for miRNAs identified as significantly dysregulated in splenic hemangiosarcoma. Target score reflects computational prediction confidence.

SUPPLEMENTARY TABLE 8 miRDB-predicted mRNA targets of differentially expressed cardiac hemangiosarcoma miRNAs. Predicted mRNA targets generated using miRDB for significantly dysregulated cardiac hemangiosarcoma miRNAs.
