# Supplementary figures and images for: MicroRNA differential expression analysis in canine visceral hemangiosarcoma formalin-fixed, paraffin-embedded tissues
Source: Front Vet Sci. 2026 Mar 17;13:1755166. doi: 10.3389/fvets.2026.1755166 (PMC13037417; doi:10.3389/fvets.2026.1755166)

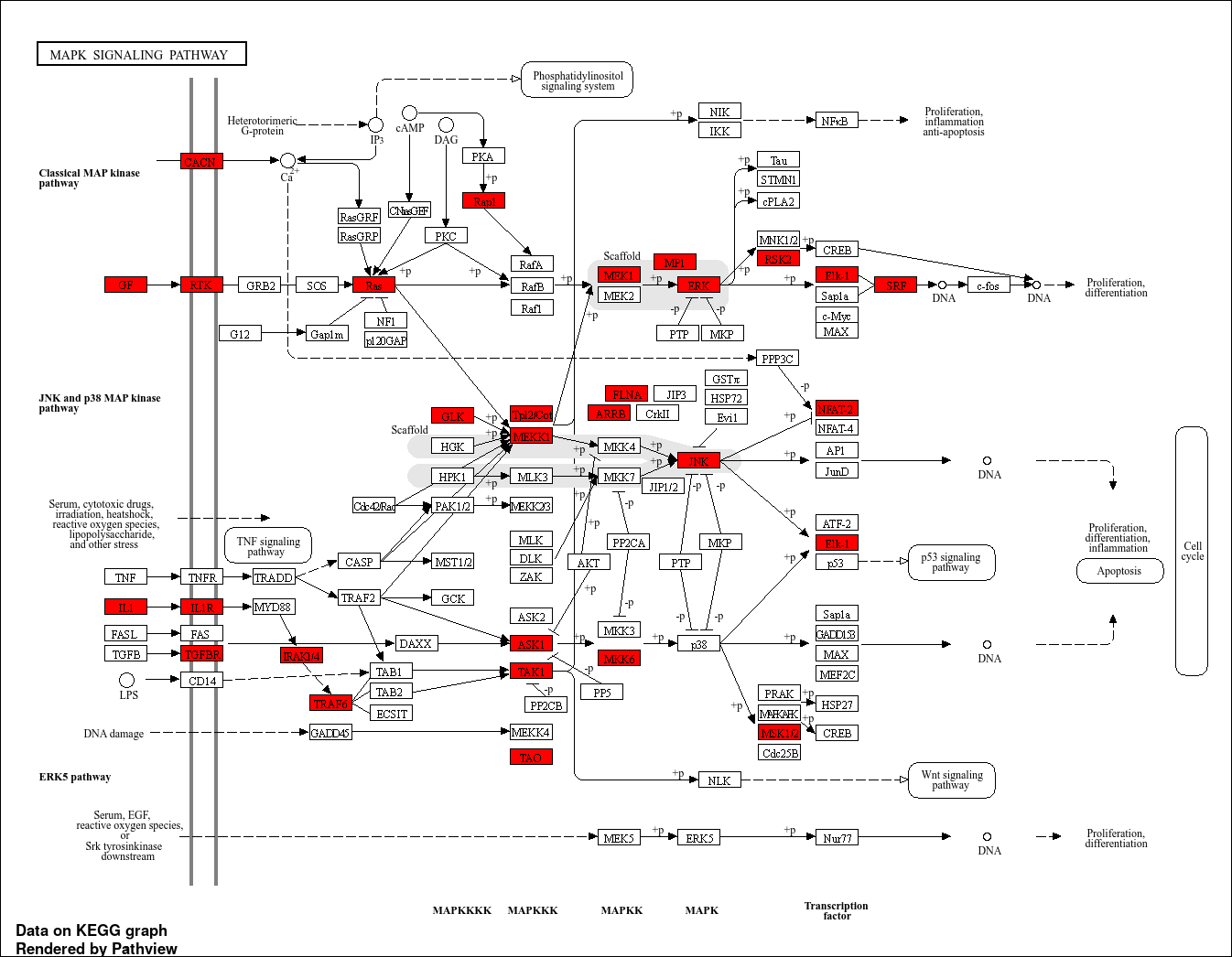

Supplement: SUPPLEMENTARY FIGURE 1 — KEGG MAPK signaling pathway enrichment of differentially expressed splenic hemangiosarcoma miRNAs. KEGG pathway map of the MAPK signaling cascade displaying predicted gene targets of significantly differentially expressed miRNAs identified in splenic hemangiosarcoma by DESeq2 analysis (adjusted p-value <0.05). Target genes predicted using miRDB are highlighted in red. [file Image_1.jpeg]

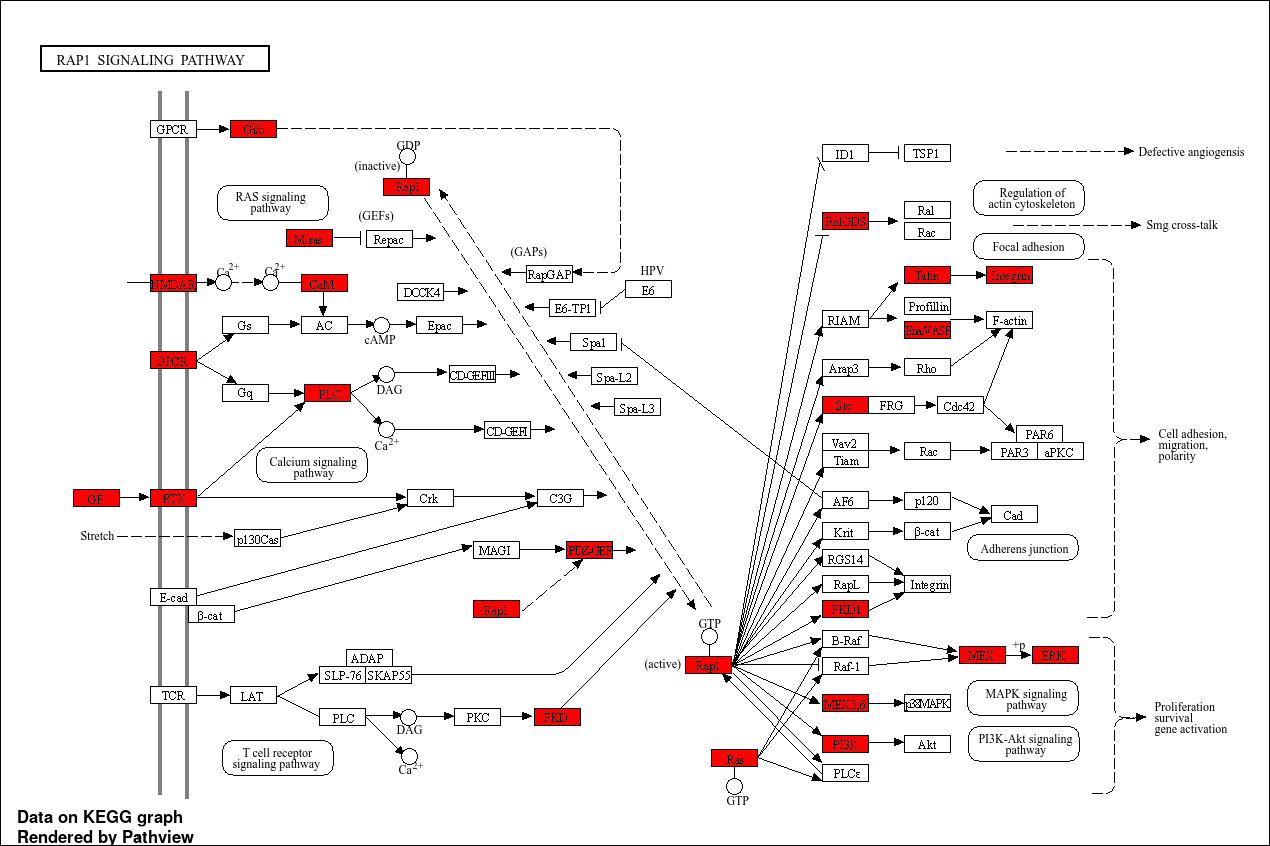

Supplement: SUPPLEMENTARY FIGURE 2 — KEGG Rap1 signaling pathway enrichment of splenic hemangiosarcoma-associated miRNAs. KEGG pathway diagram of the Rap1 signaling pathway showing predicted mRNA targets (red) of significantly dysregulated splenic hemangiosarcoma miRNAs. Predicted targets were generated using miRDB. [file Image_2.jpeg]

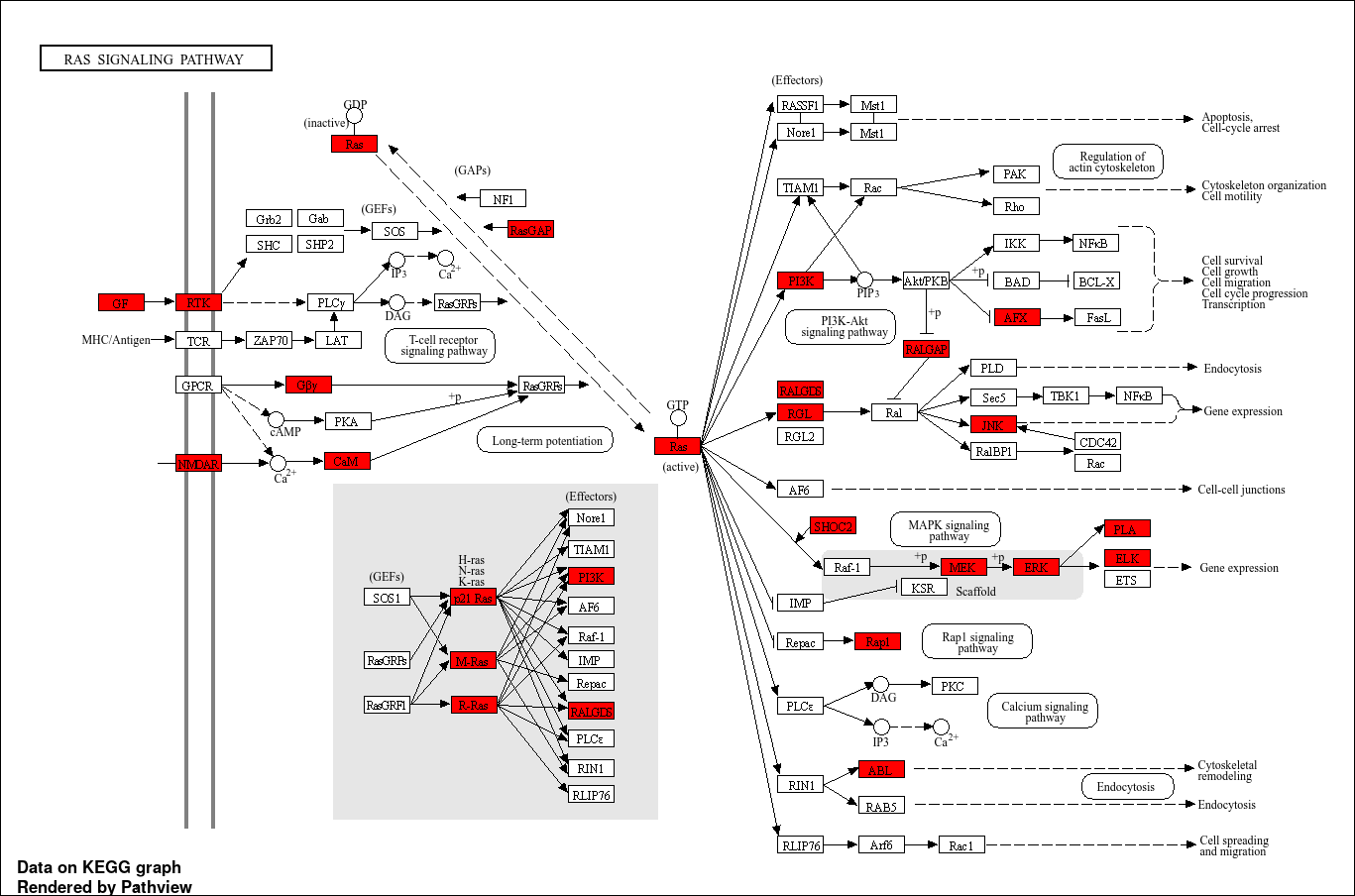

Supplement: SUPPLEMENTARY FIGURE 3 — KEGG Ras signaling pathway enrichment of splenic hemangiosarcoma-associated miRNAs. KEGG Ras signaling pathway map illustrating predicted mRNA targets (red) of differentially expressed splenic hemangiosarcoma miRNAs. [file Image_3.jpeg]

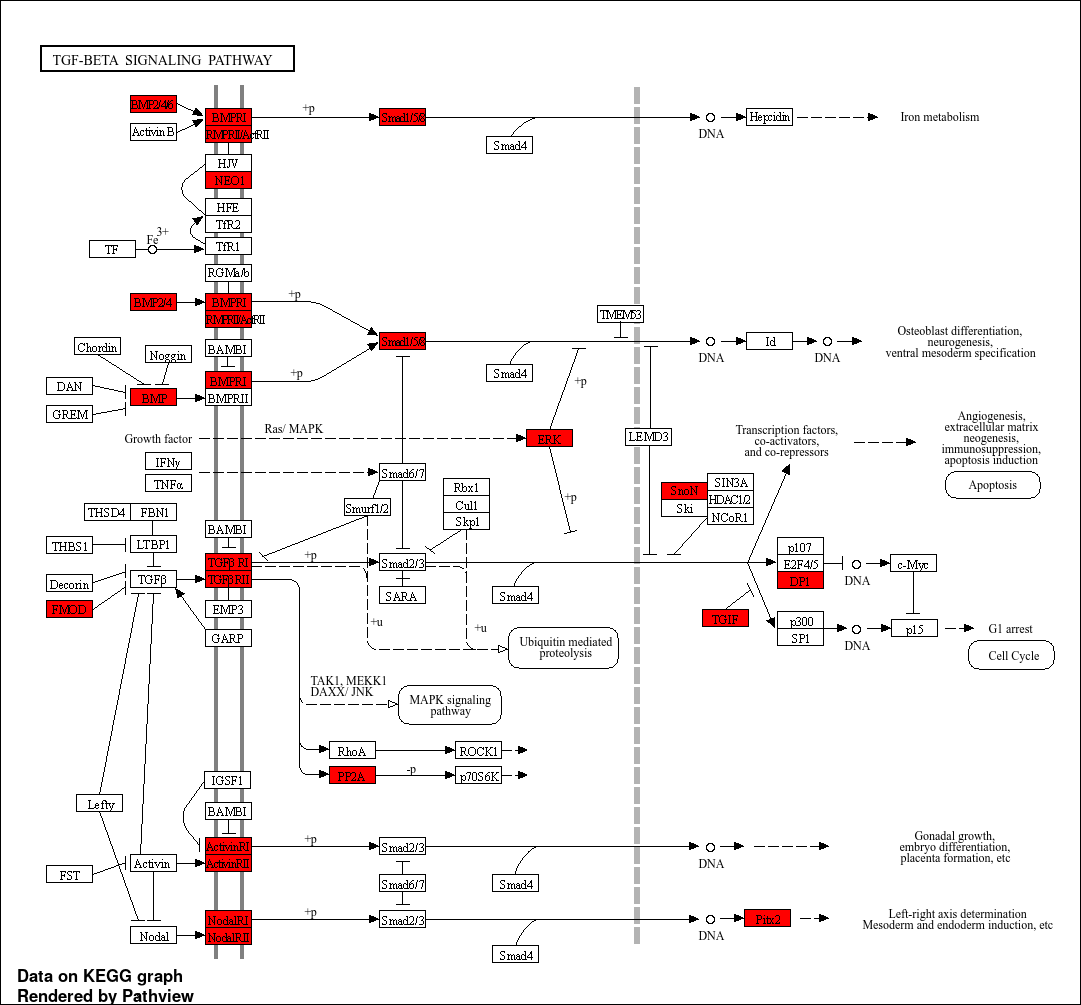

Supplement: SUPPLEMENTARY FIGURE 4 — KEGG TGF-β signaling pathway enrichment of splenic hemangiosarcoma-associated miRNAs. KEGG pathway representation of the TGF-β signaling cascade showing predicted targets (red) of significantly dysregulated splenic miRNAs. Both SMAD-dependent and SMAD-independent branches are depicted. [file Image_4.jpeg]

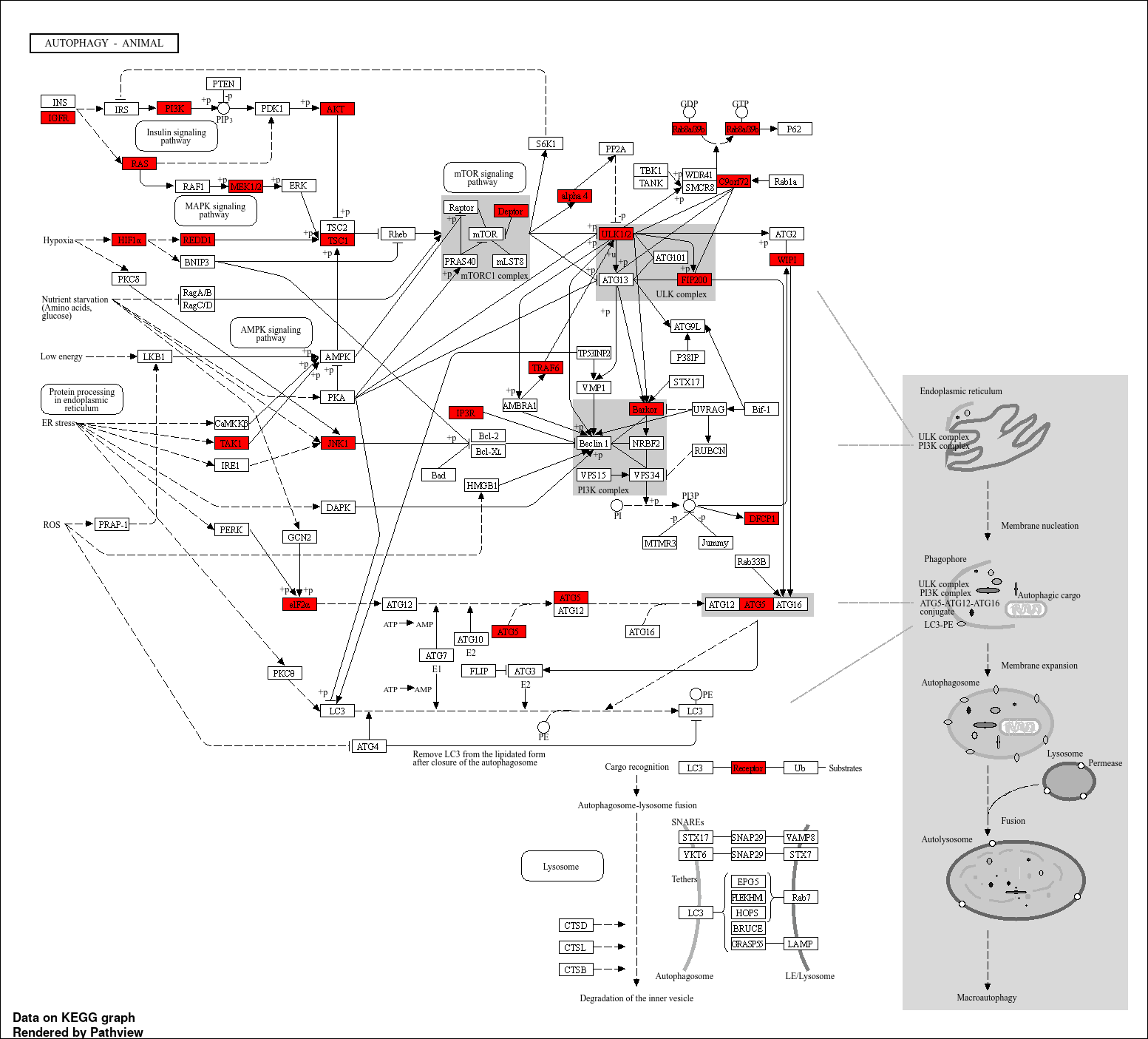

Supplement: SUPPLEMENTARY FIGURE 5 — KEGG autophagy pathway enrichment of cardiac hemangiosarcoma-associated miRNAs. KEGG autophagy pathway map displaying predicted mRNA targets (red) of significantly differentially expressed cardiac hemangiosarcoma miRNAs identified by DESeq2. Predicted interactions were obtained from miRDB. [file Image_5.jpeg]

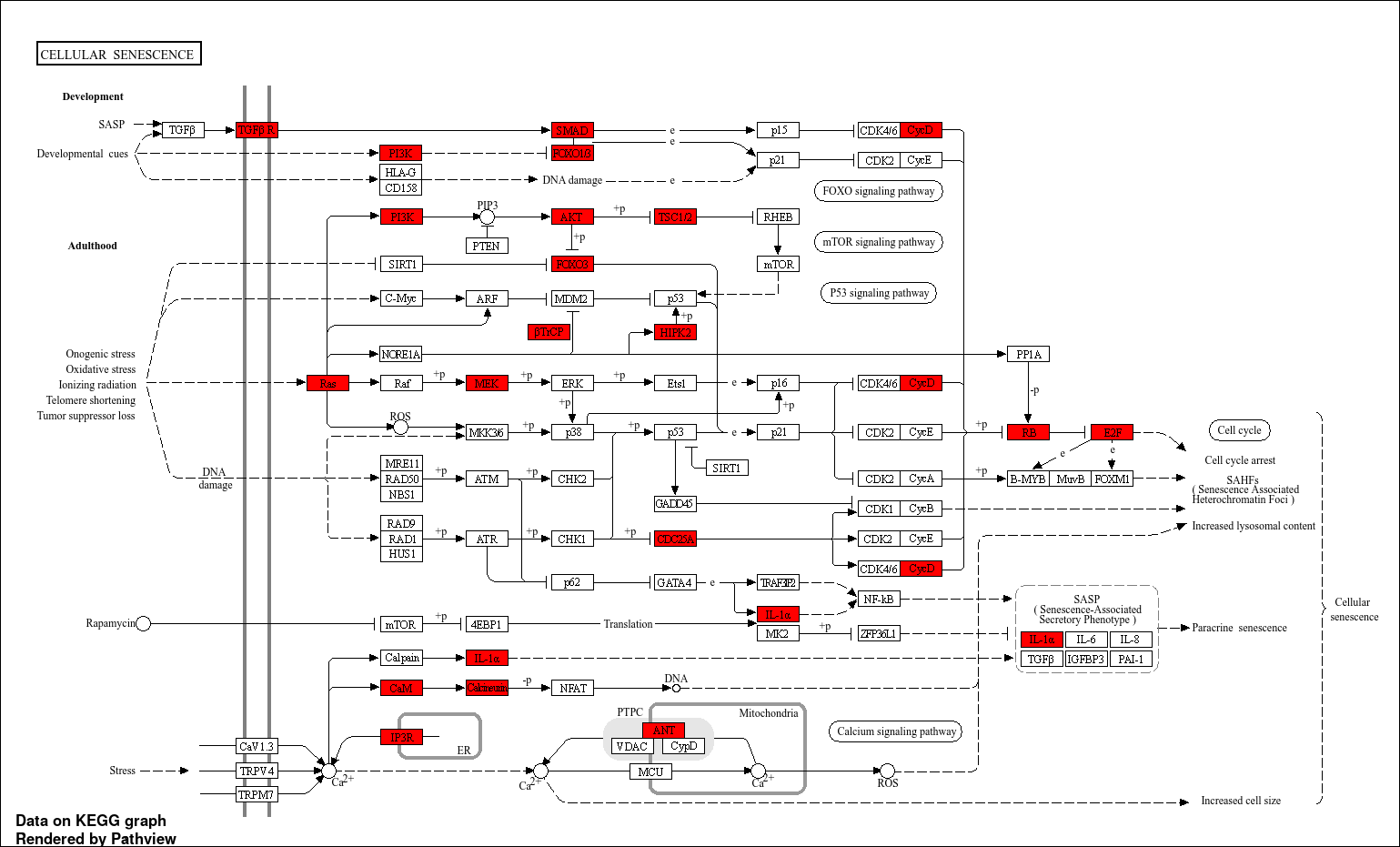

Supplement: SUPPLEMENTARY FIGURE 6 — KEGG cellular senescence pathway enrichment of cardiac hemangiosarcoma-associated miRNAs. KEGG cellular senescence pathway showing predicted targets (red) of dysregulated cardiac hemangiosarcoma miRNAs. [file Image_6.jpeg]

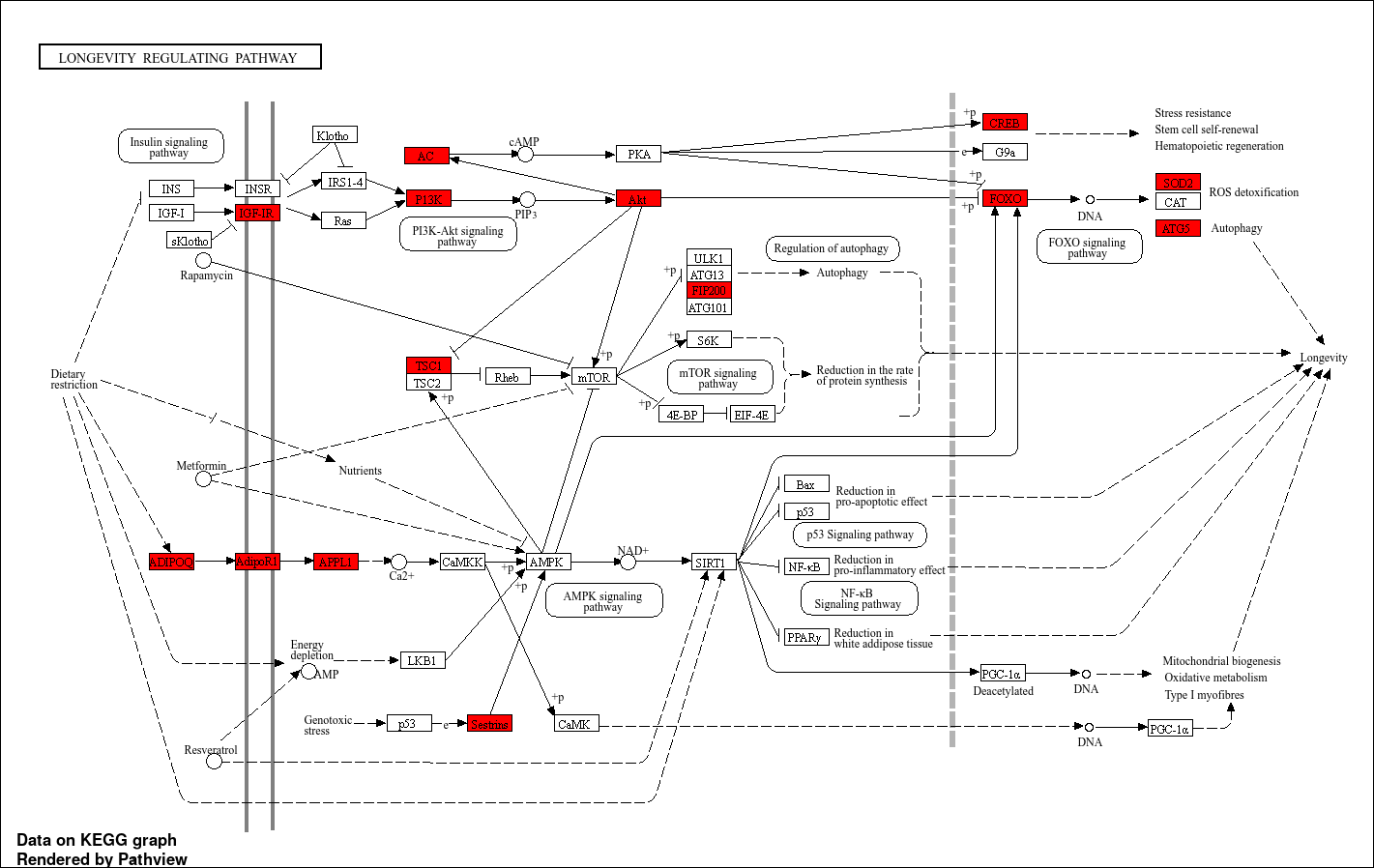

Supplement: SUPPLEMENTARY FIGURE 7 — KEGG longevity regulating pathway enrichment of cardiac hemangiosarcoma-associated miRNAs. KEGG longevity regulating pathway map illustrating predicted targets (red) of significantly differentially expressed cardiac miRNAs. [file Image_7.jpeg]

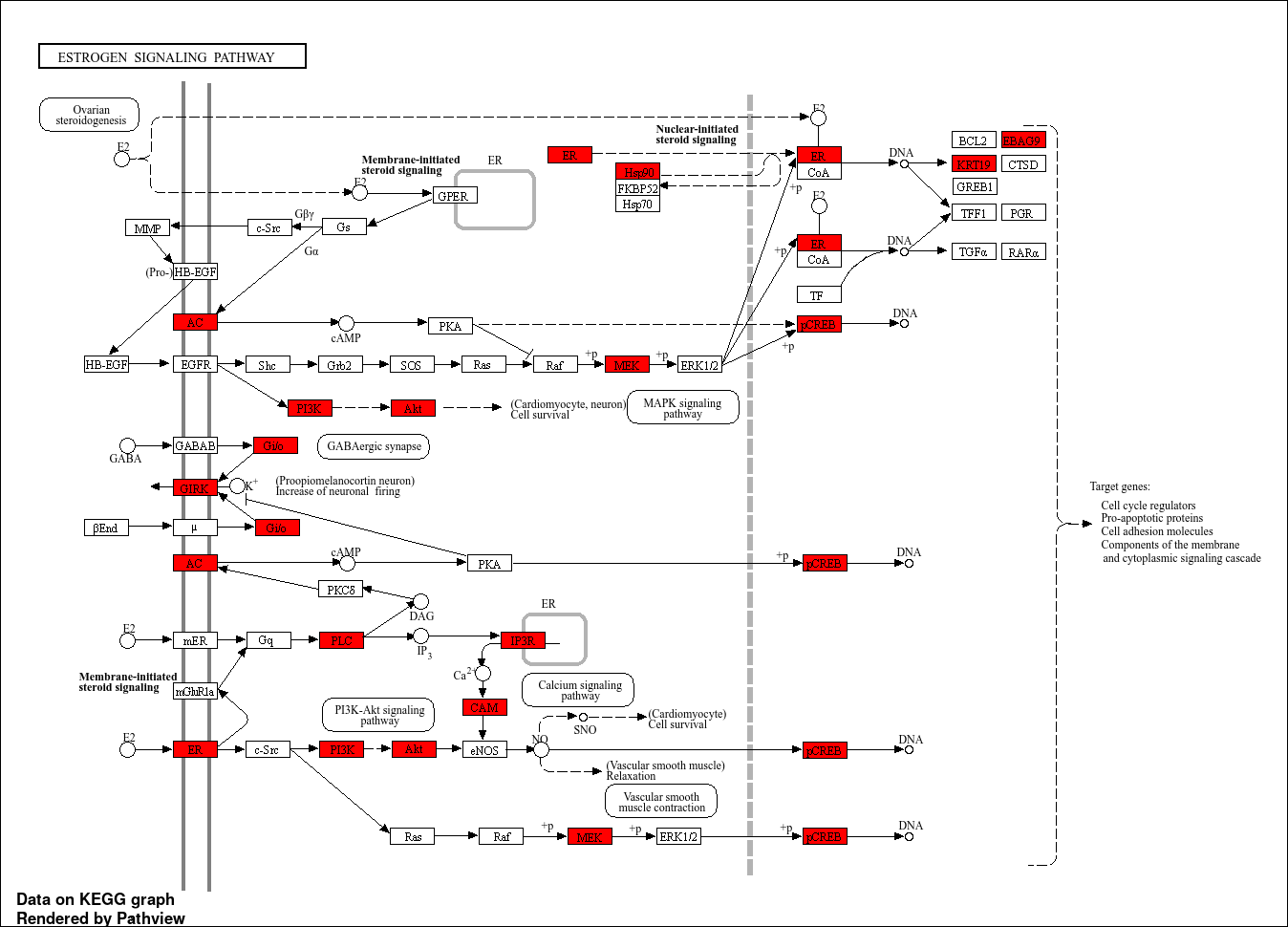

Supplement: SUPPLEMENTARY FIGURE 8 — KEGG estrogen signaling pathway enrichment of cardiac hemangiosarcoma-associated miRNAs. KEGG estrogen signaling pathway diagram showing predicted gene targets (red) of differentially expressed cardiac hemangiosarcoma miRNAs. Both genomic and non-genomic estrogen receptor signaling components are represented, including interactions with MAPK and PI3K pathways. [file Image_8.jpeg]
